# Critical illness-associated limb and diaphragmatic weakness

**DOI:** 10.1097/MCC.0000000000001135

**Published:** 2024-01-16

**Authors:** Valentine Le Stang, Nicola Latronico, Martin Dres, Michele Bertoni

**Affiliations:** a Sorbonne Université, INSERM, UMRS1158 Neurophysiologie respiratoire expérimentale et clinique; b AP-HP. Sorbonne Université, Hôpital Pitié-Salpêtrière, Service de Médecine Intensive –Réanimation (Département ‘R3S’), Paris, France; c Department of Medical and Surgical Specialties, Radiological Sciences and Public Health, University of Brescia; d Department of Emergency, ASST Spedali Civili University Hospital, Piazzale Ospedali Civili, 1, 25123 Brescia, Italy; e ‘Alessandra BONO’ Interdepartmental University Research Center on LOng Term Outcome (LOTO) in Critical Illness Survivors, University of Brescia, Brescia, Italy

**Keywords:** diaphragmatic dysfunction, ICU-acquired weakness, mechanical ventilation, muscle weakness

## Abstract

**Purpose of review:**

In the current review, we aim to highlight the evolving evidence on the diagnosis, prevention and treatment of critical illness weakness (CIW) and critical illness associated diaphragmatic weakness (CIDW).

**Recent findings:**

In the ICU, several risk factors can lead to CIW and CIDW. Recent evidence suggests that they have different pathophysiological mechanisms and impact on outcomes, although they share common risk factors and may overlap in several patients. Their diagnosis is challenging, because CIW diagnosis is primarily clinical and, therefore, difficult to obtain in the ICU population, and CIDW diagnosis is complex and not easily performed at the bedside. All of these issues lead to underdiagnosis of CIW and CIDW, which significantly increases the risk of complications and the impact on both short and long term outcomes. Moreover, recent studies have explored promising diagnostic techniques that are may be easily implemented in daily clinical practice. In addition, this review summarizes the latest research aimed at improving how to prevent and treat CIW and CIDW.

**Summary:**

This review aims to clarify some uncertain aspects and provide helpful information on developing monitoring techniques and therapeutic interventions for managing CIW and CIDW.

## INTRODUCTION

In the intensive care unit (ICU), several risk factors may induce severe weakness of the limbs and the diaphragm. These two conditions have been defined as ICU-acquired weakness (ICU-AW) [1] and critical illness-associated diaphragm weakness (CIDW) [2]. Recently, the term critical illness weakness (CIW) has been proposed as more appropriate than ICU-AW to describe the generalized weakness that affects acutely ill patients [3^▪^]. CIW, in assonance with critical illness polyneuropathy (CIP), critical illness myopathy (CIM) and CIDW, emphasizes that severe weakness is not an exclusive ICU complication. Rather it should be described as being the ‘extreme end of a spectrum of weakness that begins with any serious illness regardless of care location’ [1].

Although CIW and CIDW share common risk factors and may overlap in some patients, evidences suggest they have distinct pathophysiological mechanisms and have a different impact on outcomes [4,5]. This review aims to define better some areas of uncertainty about the risk factors, diagnosis, monitoring, and treatment of CIW and CIDW and to describe the most important new developments in recent research. 

**Box 1:**
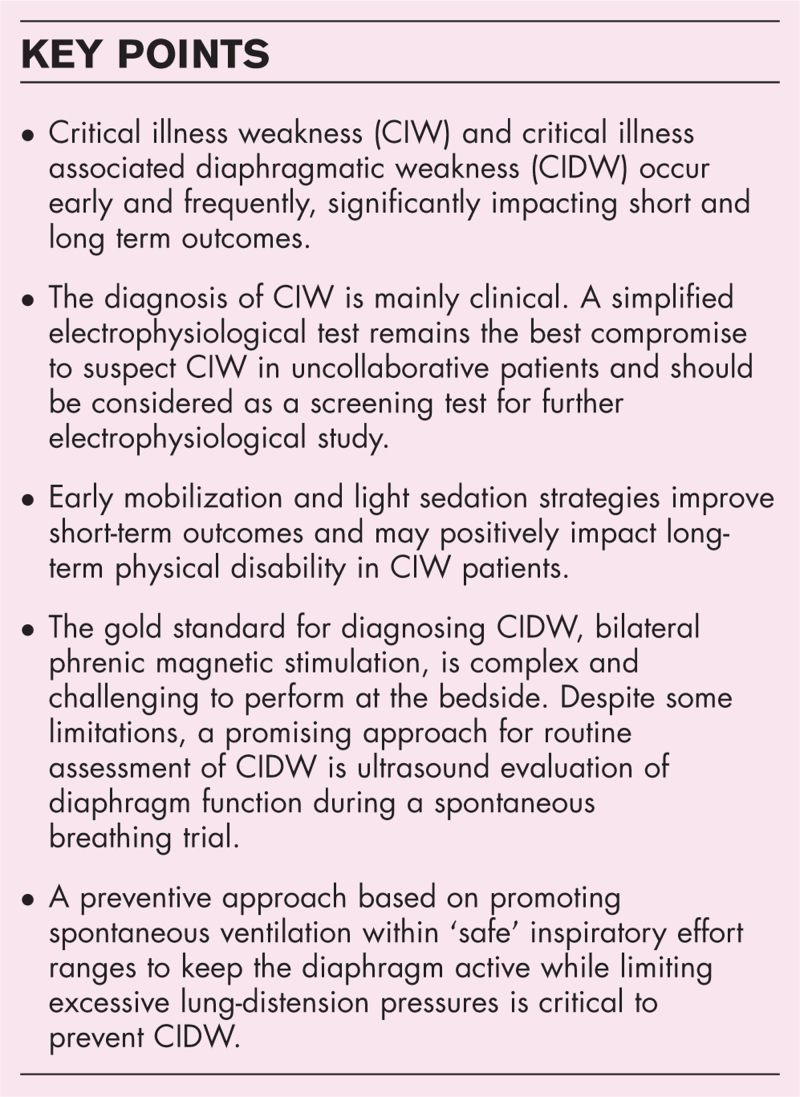
no caption available

## CRITICAL ILLNESS WEAKNESS

### Definition

In 2009, Stevens *et al.*[6] defined ICU-AW as a ‘clinically recognized weakness in critically ill patients without a plausible etiology other than critical illness’. Typically, it arises following ICU admission and occurs in conjunction with other critical illness manifestations [7]. A panel of experts in 2016 revised the ICU-AW definition to emphasize its primary link with illness severity rather than care location [1]. Recently, the term CIW has been proposed instead of ICU-AW [3^▪^].

CIW symmetrically involves all limbs, affecting proximal muscles more so than distal. Deep tendon reflexes may be reduced or unchanged; respiratory muscles are variably affected, whereas facial and extraocular muscles are generally untouched. These features and their onset after ‘exposure to critical illness’ and ICU admission distinguish CIW from other neuromuscular disorders that instead lead to ICU admission (e.g., Guillain-Barre syndrome, myasthenia gravis, other myopathies, and neuropathies) [8].

CIW may be due to a variable overlap of three distinct entities: CIP, CIM, and muscle disuse atrophy [3^▪^]. As for CIW incidence, ranges vary from 25% to 60%, depending on the characteristics of the patients studied, the time, and the methods used for its diagnosis [3^▪^,^9^], with a median incidence of 43% reported in a systematic review [10].

### Risk factors

Several risk factors are associated with CIW, with the main ones being prolonged inflammatory status [11–13], multiorgan failure, prolonged mechanical ventilation, and ICU length of stay [14]. As evidence, severity scores correlate highly with CIW [15]. Sepsis [16] and moderate to severe acute respiratory distress syndrome (ARDS) are important risk factors for CIW [15,17,18]. Emerging data suggest that acute kidney injury (AKI) may contribute to CIW involving interacting factors such as altered amino-acid metabolism, systemic inflammation, and immobility [19].

Additional ICU risk factors include hyperglycemia, a modifiable risk factor for muscle weakness [3^▪^]. Other potentially modifiable risk factors include vasopressors [20], sedatives, and selected antibiotics [21], whose harmful effects can be insidious. Corticosteroids and neuromuscular blocking agents (NMBAs) also play a role, although their long-term effects are poorly known [22,23].

Age and physical status play a role in muscle weakness conditions. Specifically, older age and frailty before acute disease onset are highly correlated with CIW [15].

### Diagnosis and monitoring

CIW is diagnosed clinically in all cooperative patients by manually assessing muscle strength. The Medical Research Council sum score (MRCss) is considered the gold standard for CIW diagnosis [3^▪^]. The assessment of 12 limb muscle groups is necessary to diagnose CIW, defined by an MRCss less than 48. An MRCss less than 36 identifies a more severe form of CIW; however, a milder reduction of limb muscle strength with an MRCss of<55 is associated with increased long-term morbidity and mortality [24^▪▪^]. Despite its high inter-rater reliability, MRCss can be challenging because it is operator-dependent, time-consuming, and requires expertise [25,26]. Handgrip Dynamometry (HGD) is a simple, repeatable test that assesses dominant hand strength in cooperative patients. Dominant HGD values below 11 kg in males and 7 kg in females strongly suggest the diagnosis of CIW and can be used to rapidly detect CIW, which should be confirmed with MRCss [3^▪^,^16^].

**FIGURE 1:**
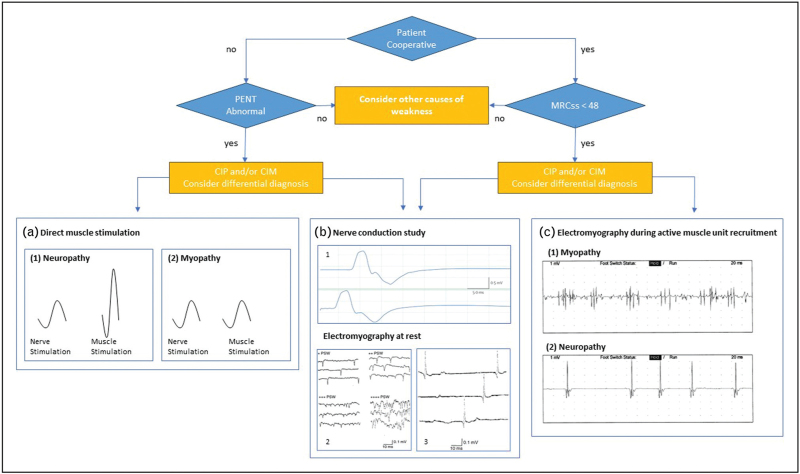
Diagnostic algorithm for critical illness weakness, polyneuropathy and myopathy. (b1) Adapted from [27]. In critical illness polyneuropathy (CIP), nerve conduction studies show a reduction in the amplitude of both compound muscle action potential (CMAP) and sensory nerve action potential (SNAP, not shown). In critical illness myopathy (CIM), CMAP is reduced while SNAP remains normal. (b2, b3) At rest electromyography (EMG), various degrees of positive sharp waves (b2) and fibrillation potentials (b3) may be present in both CIP and CIM. (c) With cooperative patients, motor unit potentials at EMG are large in amplitude and have a long duration in CIP, whereas in CIM, they are low in amplitude and short; both are highly polyphasic. (a) Adapted from [96]. Direct muscle stimulation (DMS) can differentiate CIP and CIM in uncooperative patients: in CIM, CMAP reduction occurs after both conventional nerve stimulation and DMS, whereas in CIP CMAP reduction occurs only after conventional nerve stimulation. MRCss, medical research council sum score; PENT, peroneal nerve test. Reference [27,96].

As MRCss and HGD require full patient cooperation, other nonvolitional tests can be used in uncooperative patients. Electrophysiologic testing of peripheral nerves and muscle (nerve conduction study and electromyography, NCS-EMG) can reveal electrical neuromuscular abnormalities before weakness occurs and are essential not only to differentiate CIP from CIM [27], but also to differentiate CIP and CIM from other acute neuromuscular disorders [3^▪^]. Among NCS, the simplified peroneal nerve test (PENT) can detect a reduction of the compound muscle action potential (CMAP) amplitude of the common peroneal nerve and has been validated in two multicenter studies as a screening method to identify CIM or CIP with 100% sensitivity and high specificity [28,29]. The altered electrical excitability of nerves and muscles is associated with increased long-term mortality independent of muscle weakness. PENT remains the best compromise to diagnose CIP and/or CIM in all uncooperative patients, in whom it should be considered a screening test for further electrophysiological study [3^▪^]. A complete electrophysiological investigation (NCS-EMG) allows for the complete evaluation of peripheral nerve and muscle function and enables the differential diagnosis of CIM and CIP but requires specialized expertise and is usually chosen wherever a specific etiological diagnosis is required (Fig. 1) [16].

It is worth noting that disuse atrophy causing CIW may manifest without electrophysiologic changes and may go undetected in uncooperative patients [3^▪^]. Muscle biopsy can be required in these cases to clarify the pathological diagnosis and provide further prognostic information. For example, muscle necrosis usually indicates a poor prognosis, whereas a tick filament myopathy with loss of myosin filaments is associated with better functional recovery [27]. Biopsy of the motor nerve to the *gracilis* muscle may be considered for research purposes or complex differential diagnoses [30].

Nerve and limb muscle ultrasound is a noninvasive, bedside technique that can be used for daily monitoring of limb muscle changes [31]. However, further research is needed to validate it [32]. Nonvoluntary electrical or magnetic supramaximal twitch nerve stimulation is promising [33,34]. Combined use of ultrasound and magnetic twitch assessment of the quadriceps shows that thickness often remains in the normal range when strength is severely reduced [35]. Clinical and electrophysiological features of CIP and CIM are summarized in Fig. 1.

## CRITICAL ILLNESS ASSOCIATED DIAPHRAGMATIC WEAKNESS

### Definition and risk factors

#### What is already known

Ventilator induced diaphragm dysfunction (VIDD) is a term derived from experimental studies in which the diaphragm of animals selectively exposed to invasive mechanical ventilation developed a decrease in the force generated by the stimulation of diaphragm fibers *in vitro*[36]. In ICU patients, it is impossible to isolate mechanical ventilation's effects on the diaphragm and other risk factors (sepsis, shock, multiorgan failure), leading to the term critical illness associated diaphragmatic weakness [2]. CIDW is associated with a time-dependent decrease in diaphragm generating pressure in critically ill patients. As well as for the CIW, CIDW may result from muscular impairment or nerve impairment (axonopathy) or both [37]. Of note, diaphragm dysfunction may be caused by direct damage to the phrenic nerve after cardiac or thoracic surgery. CIDW is frequent and largely underdiagnosed in the ICU, with a prevalence estimated between 60% and 80% [2].

#### What is new

Studies have investigated the relationship between respiratory muscles activity during mechanical ventilation and the occurrence of CIDW. Low diaphragm activity is associated with diaphragm thinning, a surrogate estimate of diaphragm atrophy [38]. Excessive diaphragm activity, as estimated by high diaphragm thickening fraction (TF) on ultrasound examination, is associated with increased diaphragm thickness suggesting diaphragm injury (and not hypertrophy). Importantly, both increased and decreased diaphragm thickness are associated with a reduced probability of successful weaning [39]. These results suggest that, beyond the risk of respiratory muscles unloading induced by mechanical ventilation, excessive inspiratory efforts during invasive and noninvasive respiratory supports may harm the diaphragm (insufficient unloading). Excessive or inappropriate inspiratory efforts are frequent in case of patient-ventilator asynchronies and may be associated with CIDW [40,41^▪▪^]. In animals breath stacking, and reverse triggering were associated with eccentric contractions of the diaphragm leading to a significantly reduced diaphragm force generation and histological abnormalities [40]. Whether inappropriate ventilator settings may lead to CIDW is a relevant question. Positive end-expiratory pressure (PEEP) induces changes in diaphragm geometry, especially muscle shortening, and decreases in vivo diaphragm contractile function [42]. In veno-venous extra-corporeal membrane oxygenation (VV-ECMO) ARDS patients, an higher level of PEEP before ECMO implantation was associated with CIDW [43]. Sepsis is associated with a severe but reversible CIDW and improved diaphragm function is associated with better survival [44].

#### Monitoring the diaphragm function: what is new?

**FIGURE 2:**
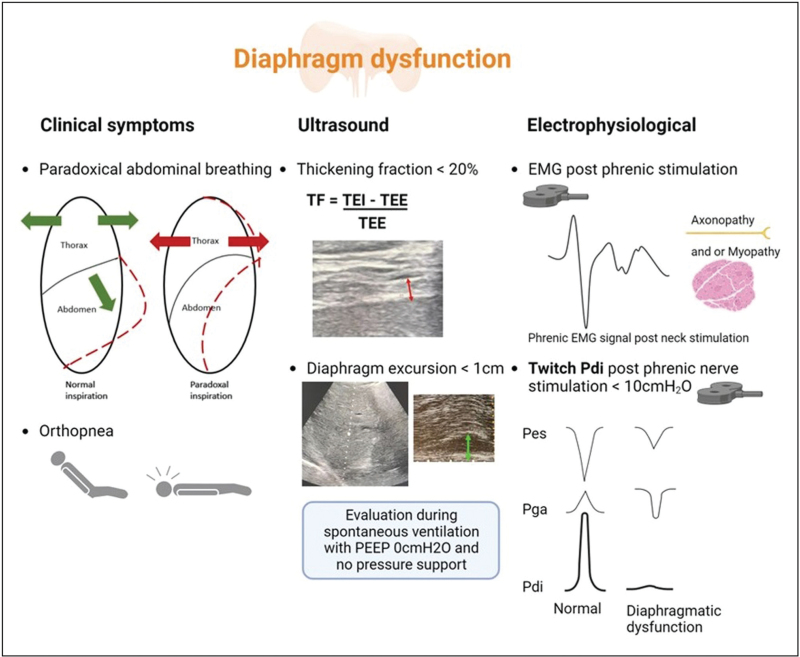
Diagnostic features of critical illness-associated diaphragmatic weakness in intensive care patients. EMG, electromyography; *P*_di_, transdiaphragmatic pressure; PEEP, positive-end expiratory pressure; *P*_es_, esophageal pressure; *P*_ga_, gastric pressure; TEE, end expiratory thickness; TEI, end inspiratory thickness; TF, thickening fraction.

The reference method to evaluate the diaphragm function in the ICU is the bilateral anterior magnetic phrenic nerve stimulation technique that measures the diaphragm pressure generating capacity during a standardized stimulation. This method, however, is complex and not easily implemented at the bedside, which explains the growing interest in diaphragm ultrasound (DU). Although it is a noninvasive and easily accessible technique, DU does not directly measure the diaphragm function, i.e. the transdiaphragmatic pressure (*P*_di_). Instead, DU provides a surrogate measure of diaphragm activity, such as the TF, the diaphragm excursion (DE) and the diaphragm velocity [45]. DU is operator-dependent which is associated with an inherent bias related to the precision of the measurements. The clinical value of diaphragm TF has recently been challenged since it correlates weakly with *P*_di_[35,46,47], suggesting that diaphragm TF on DU may be a poor surrogate of diaphragm function. Instead, diaphragm TF remains a valuable measurement for predicting weaning outcome and assessing the level of inspiratory effort since it behaves as one of the components of the respiratory capacity/load balance. Diaphragm stiffness can be assessed using shear wave elastography that provides additional information about the diaphragm structure [48,49]. Similarly, tissue Doppler imaging is a complementary tool to assess diaphragm function [50,51]. Diagnostic features of CIDW are summarized in Fig. 2.

## IMPACT ON OUTCOME

### Critical illness weakness: what is new?

One consequence associated with CIW pertains to poorer short-term outcomes. In weak patients, dysphagia and impaired cough predispose to prolonged duration of mechanical ventilation [52], making CIW highly correlated with delayed weaning and extubation failure [4,53,54^▪^]. However, causality needs further exploration as prolonged mechanical ventilation induces immobility, a proven risk factor for CIW, making this correlation difficult to interpret. CIW is also associated with prolonged ICU and hospital stay, which increases the risk of ICU and hospital mortality [3^▪^,^55^,^56^].

CIW at ICU discharge is associated with an increased risk of prolonged muscle weakness and physical disability and is associated with long-term mortality. CIP is the leading cause of persistent disability and poor functional status, while CIM has a better prognosis. Patients who are primarily affected by CIP are more likely to have a slower or no recovery and to have an increased mortality rate [28,29]. Likewise, CIW patients have a higher rate of ICU readmission and an increased risk of late death [24^▪▪^,^57^]. Functional impairment may persist throughout life [15,58]. Physical disability can be associated with cognitive impairment, depression and chronic pain, making it difficult to return to daily living and work activities. Chronic pain, either nociceptive, neuropathic, or neoplastic pain, is frequent in ICU survivors, varying from 14% to 77% depending on the population assessed, the tool used to measure pain, and the time point when pain was assessed in various published series [59]. It can be highly disabling, causing moderate to severe limitations in activities of daily living in >50% of patients. Neuropathic pain is primarily related to the degeneration of small nerve fibers [60]; in CIM, muscle inflammation, decreased muscle tone, and immobilization also play a role [61,62].

These sequelae severely impact the quality of life of ICU survivors and have been described as part of the Post-Intensive Care Syndrome (PICS) [63].

### Critical illness associated diaphragmatic weakness: what is new?

CIDW is frequent in intubated patients and is associated with prolonged duration of mechanical ventilation [53] and poor prognosis [64]. A DE lower than 1.2 cm, is associated with an increased risk of intubation [65]. After intubation, CIDW has a negative impact on successful weaning from mechanical ventilation. DE or TF measured during a spontaneous breathing trial (SBT) are good predictors of extubation success although the TF appears to be less sensitive than DE [66–68]. A systematic review reported that DE above 10 mm and diaphragm TF up to 29% perform well in predicting successful extubation [69]. Of note, two studies found no association between a low diaphragm TF and extubation failure during tidal breathing but not during SBT [5,70]. The same was reported for DE in another study [71]. This highlights the importance of evaluating the diaphragm during a challenge test. DE measurements during assisted mechanical ventilation are consequently overestimated because the patient's diaphragmatic contraction is added to the passive excursion generated by the ventilator in pressure support mode [72]. Diaphragm dysfunction is also common following bilateral lung transplantation and is associated with difficult weaning. In these cases, TF and neuro-ventilatory efficiency have similar accuracy for predicting ventilator weaning success, demonstrating an inverse relationship with duration of ventilation [68].

In contrast with CIW, which is associated with long-term outcomes, CIDW evaluated on the day of the first SBT is not associated with long-term survival after ICU discharge [73]. As a possible interpretation, diaphragm function is a critical determinant of weaning outcome, but once patients have been weaned off the ventilator, longer term prognosis is mainly determined by the presence of CIW.

**FIGURE 3:**
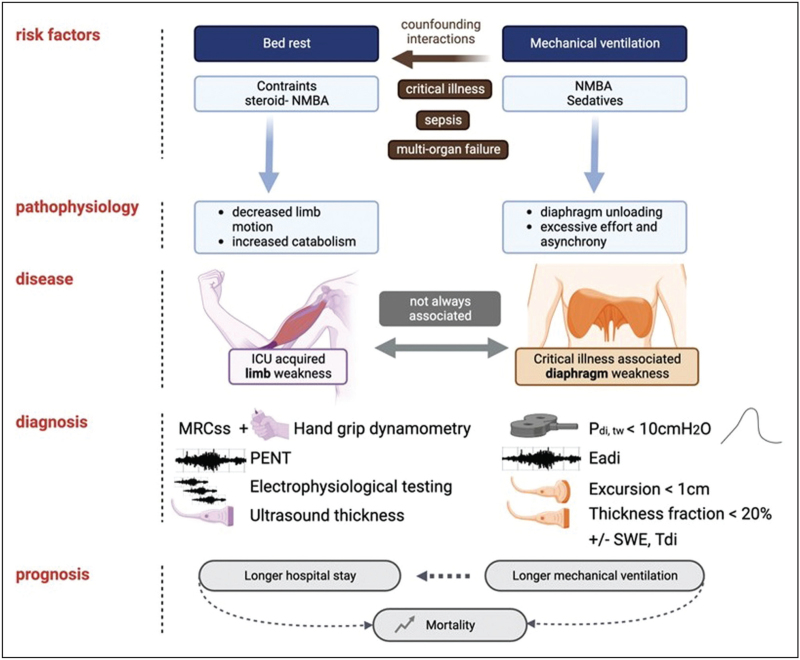
Schematic presentation of critical illness-associated limb and diaphragm weakness. Eadi, diaphragmatic electromyography; ICU, intensive care unit; MRCss, Medical Research Council sum score; NMBA, neuromuscular blocking agents; *P*_di,tw_, twitch trandiaphragmatic pressure; PENT, peroneal nerve test; SWE, shear wave elastography; Tdi, tissue Doppler imaging.

Figure 3 summarizes CIW and CIDW risk factors and diagnostic tests.

## MANAGEMENT

### Critical illness weakness: what is new?

As of yet, there are no specific therapies to treat CIW. Strategies based on aggressive management of critical illness and minimization of ICU risk factors may be beneficial [1].

Immobility is a known risk factor for CIW and should be avoided as much as possible. Mobilization should be performed in all ICU patients, but the level and timing are still uncertain. Early mobilization starts when the patient's condition has stabilized and is effective in achieving higher mobility levels and better functional status at hospital discharge, but the heterogeneity of the studies, in terms of populations and interventions, leaves the efficacy and safety of early mobilization unsolved [74–76]. Ideally, a ‘personalized’ dose of mobilization tailored to the patient's needs would be safe and effective in reducing short-term disability. Rehabilitation protocols should not stop at ICU discharge and should indeed continue in the general ward and after discharge from the hospital, involving multidisciplinary and multiprofessional teams and family members [3^▪^].

Implementing light sedation strategies should be encouraged. A daily plan to reduce and interrupt sedation (ABCDEF bundle) has been shown to prevent delirium and immobility and promote more rapid weaning from mechanical ventilation [77,78]. Minimizing sedation with appropriate pain relief may increase patient comfort and provide a better rehabilitation and occupational plan.

Digging deeper into food intake, severe hyperglycemia should be avoided [79,80], and nutritional status should be closely monitored. ESPEN guidelines confirmed early enteral nutrition (EN) as mandatory, while early parenteral nutrition (PN) should be avoided and limited to selected patients unable to receive EN [80]. Overnutrition and undernutrition have been shown to be deleterious, and energy goals should be reached within the first seven ICU days. Regarding protein supplementation, in the early phase, the hypercatabolic state renders the muscle unable to benefit from amino acids [81]. However, recent studies suggest that protein supplementation used in conjunction with mobilization and at a later ICU stage may be beneficial [82,83].

Not least, the use of some drugs during the acute stage of disease should be minimized. Corticosteroids are associated with myopathy and CIW and NMBAs cause patient's immobilization [23] and should be discontinued as early as deemed tolerable.

Recent promising animal studies suggest that stem cell engrafting may stimulate muscle regeneration and limit damage in sepsis-related CIW [84]. Further studies are needed to expand this field.

### Critical illness associated diaphragmatic weakness: what is new?

Managing CIDW can be done through preventive or curative approaches [85]. The preventive approach is based on promoting spontaneous ventilation within ‘safe’ ranges of inspiratory efforts to maintain the diaphragm active (and minimize the risk of atrophy) while limiting excessive lung-distending pressures [86]. De Vries *et al.*[87] reported a protocol to provide lung and diaphragm protective ventilation, where the Pdi was maintained between 3 and 12 cmH_2_O, and the tidal volume and plateau pressures were maintained between 4 and 10 ml/kg ideal body weight and, respectively, below 30 cmH_2_O. The proportions of breaths within the target range of diaphragm effort were higher for patients in the intervention group. They also validated occlusion maneuvers to estimate lung stress and respiratory muscle effort [88^▪^]. This may help implement careful monitoring and delivering of lung and diaphragm protective ventilation at the bedside. Another possibility would be to artificially titrate the inspiratory effort in sedated patients using phrenic nerve stimulation [89]. Recent studies have reported promising technological developments that may be relevant in critically ill patients [90–92]. The curative approach is to improve the contractile force of the diaphragm in case of weaning failure. Several options have been tested, such as inspiratory muscle training, drugs or phrenic nerve stimulation [93,94], yet none have shown favorable effects on relevant outcome measures. The presence of CIDW sometimes contributes to the decision making process of tracheostomy in patients with several failed weaning attempts. However, it is noticed that many patients can be successfully separated from the ventilator despite the presence of diaphragm weakness [53,95]. In addition, tracheostomy is associated with potential complications. Last, weaning failure is often multifactorial. Therefore, it seems important to carefully rule out (and potentially treat) other risk factors of weaning failure and to weigh thoroughly the benefits and risks of the tracheostomy at the individual level. Regardless of the function of the diaphragm, clinicians might keep in mind that control of the upper airways is an essential determinant of extubation outcome.

## CONCLUSION

CIW and CIDW are common and serious complications in critically ill patients, often occurring alongside severe illness. While they share some risk factors, they may have distinct underlying causes. Early diagnosis and rapid establishment of appropriate treatments are crucial to improving short-term outcomes and may positively impact long-term physical impairments.

## Acknowledgements

*None.*

### Financial support and sponsorship

*None.*

### Conflicts of interest

*There are no conflicts of interest.*

## REFERENCES AND RECOMMENDED READING

Papers of particular interest, published within the annual period of review, have been highlighted as:

▪ of special interest
▪▪ of outstanding interest
